# The Impact of Artificial Intelligence in Enabling Digital Innovation/ The Mediating Role of Digital Transformation: A Survey Study Opinions of a Sample for Faculty Members at University of Ninevah

**DOI:** 10.12688/f1000research.173517.2

**Published:** 2026-07-08

**Authors:** MOHAMMED TALLAL MOHAMMED, Raghad Osama Jarallah, MOATASEM HOOD M. SALIH, Adel Mohammed Abdullah, Nizar Siddeek Al-gahwachi, WSAM HASAN FATHI

**Affiliations:** 1 Software Department, Information Technology College, Ninevah University, Mosul, Nineveh Governorate, Iraq; 2 Law college, Ninevah University, Mosul, Nineveh Governorate, Iraq; 3 Law college, Ninevah University, Mosul, Nineveh Governorate, Iraq; 4 Department of Industrial Management, College of Administration and Economic, University of Mosul, Mosul, Nineveh Governorate, Iraq; 5 Department of Economics, College of Administration and Economic, University of Mosul, Mosul, Nineveh Governorate, Iraq; 6 Department of Administrative and Financial Affairs, Ninevah University, Mosul, Nineveh Governorate, Iraq

**Keywords:** Artificial Intelligence, Digital Innovation, Digital Transformation, Higher education

## Abstract

**Background:**

Digital transformation, driven by artificial intelligence (AI) and digital innovation, has become a cornerstone for improving educational quality and administrative efficiency in higher education institutions. However, universities in developing countries like Iraq face significant challenges, including inadequate infrastructure, gaps in digital literacy, and resistance to change that hinder the effective integration of these technologies.

**Method:**

This study used both descriptive and quantitative analysis using statistical tools represented by SPSS and Amos, tables and graphs. Data was collected from 206 faculty members working at the University of Ninevah using an electronic questionnaire after it was evaluated by experts in the field of artificial intelligence and digital transformation.

**Results:**

The results of the statistical analysis showed that artificial intelligence has a positive impact on enabling digital innovation in universities. The study also revealed that digital transformation played a key mediating role in analyzing the relationship between artificial intelligence and digital innovation. Thus, the four hypotheses were confirmed, reflecting the validity of the hypothesized model and highlighting the importance of digital transformation as a strategic mechanism for improving the efficiency of artificial intelligence technologies and supporting digital innovation in universities.

**Conclusion:**

Our study concludes that digital transformation, which supports enhanced interaction between artificial intelligence and digital innovation, is key to improving the quality of education and management ethics in Iraqi higher education institutions. This leads to better teaching practices, more engaged students, enhanced collaboration, and support for scientific research. However, weak infrastructure support, a prevailing digital culture, and some resistance to change pose real challenges to its implementation. Despite these obstacles, digital transformation remains a top investment priority for Iraqi higher education institutions to maximize the direct benefits of artificial intelligence and digital innovation.

## 1. Introduction

The world today is witnessing rapid developments and unprecedented competition among higher education institutions across the globe using artificial intelligence (AI) and digital technologies, which have become a vital and essential part of the educational process. ( Alshammari et al., 2025) added that artificial intelligence and metaverse offer numerous benefits in higher education, benefiting both students and teachers. For students, these benefits include tracking their academic work, writing reports, generating ideas, and communicating with academic bodies within the educational institution. On the other hand, the benefits for teachers, as ( Alshammari et al., 2026) indicated, can be summarized as generating research ideas, reframing statements, linking variables in practical research, formulating questions, creating algorithms, interacting with students, and developing scientific concepts. When used correctly, AI can facilitate lectures and develop and simplify course material for students. AI also has other capabilities, such as monitoring and supervising student performance and facilitating their diverse academic needs, ultimately contributing to student development and the expansion of their academic horizons ( Sajja et al., 2024; Wang et al., 2024). Proficiency in digital technology allows instructors great flexibility in selecting the educational applications most appropriate for the student’s level in the classroom and accelerates greater adaptation to the academic and educational environment ( Behera et al., 2025). Digital innovation contributes to the creation and updating of educational programs to suit the labor market of the country or region concerned with study and scientific research. It develops scientific and research cadres and raises the level of university graduates, preparing them to be competent future graduates ( Priyadi & Arwani, 2024; Zawacki et al., 2019). Digital innovation used in personalized education analyzes data and facilitates the process of predicting students’ educational needs. It facilitates academic exchange and raises educational quality ( Selwyn and Facer, 2021). Faculty participation in the application of these technologies is an important element in linking technical capacity with the real improvement of educational outcomes. This is important for understanding the dynamics of the relationship between AI, digital transformation, and digital innovation in the education sector ( Harper, 2021; Prabowo and Bandur, 2022).

From the above, an important question arises: How can artificial intelligence and digital transformation be employed to achieve digital innovation in the Iraqi educational environment, especially at the higher education level in Iraqi universities? Is the path to this through developing the teaching staff? Are they capable of absorbing and understanding modern technology and clearly conveying it to students? Do we first need a sound scientific and educational environment, including a correct digital transformation process? The following question stems from the main concern of the study: What is the impact of AI on DI at the University of Ninevah, and how does digital transformation act as a mediator between students and their educational and administrative goals.

The importance of this research lies in providing a comprehensive understanding of issues related to academic decision-making, which will help them develop modern strategies that contribute to developing digital infrastructure, redesigning curricula, and employing innovative AI-based educational technologies. Despite the significant development in global literature related to the subject of this study, these studies were conducted in higher education contexts characterized by institutional stability technological advancement, and ample resources. In contrast, the context of higher education in Iraq is characterized by different conditions, including administrative challenges, varying levels of digital infrastructure, and the impacts of economic and social transformations. This highlights the need to study this topic within the local context to bridge the research gap. This research also contributes to enhancing the capabilities of experienced professors in teaching students about artificial intelligence and increasing their numbers, facilitating interaction between students and faculty members and contributing to the creation of an advanced, effective, and long-term educational environment.

This research aims to achieve several objectives, including: analyzing the impact of artificial intelligence on digital innovation at the University of Ninevah; studying the role of digital transformation in this impact; and presenting results and proposals that will enhance administrative and academic performance, improve the quality of education, and create an innovative and pioneering educational environment that keeps pace with global developments.

## 2. Research model

The proposed research model explains the direct and mediating relationships between the study variables, as it is assumed that artificial intelligence (AI) directly affects digital innovation (DI) and digital transformation (DT), and it is assumed that artificial intelligence indirectly affects digital innovation through digital transformation (DT) as the mediating variable. This model is consistent with the conceptual framework that was developed in the literature.

## 3. Literature review

Study ( Al-Taai et al., 2025) is distinguished by its novelty and use of artificial intelligence at the level of Iraqi universities. One of the most important findings reached by the researchers is that: “The use of these artificial intelligence technologies had a significant impact on developing education, especially blended learning, and led to an improvement in the quality of academic performance.” This positive improvement is dependent on increased student engagement and an active learning process. The study also emphasized the importance of cooperation in using digital transformation and mastering it in monitoring student achievement and evaluating the effectiveness of smart education systems, especially in the applied aspect. Meanwhile, ( Aldulaimi et al., 2018) discussed the idea that: “Successful implementation of digital transformation based on a sound strategic approach to technological management and providing an appropriate sustainable digital learning environment” is the path to achieving sustainable success in the educational process with all its various elements. The researchers, ( Abdullah and Ahmed, 2024), also emphasized that “the strategies adopted by the Ministry of Higher Education and Scientific Research during digital transformation had a significant impact on developing and improving knowledge leadership practices,” which enhanced the effectiveness of knowledge management and improved the quality of education. The researcher ( Wahib, 2023) emphasized “the need to intensify training programs on digital learning platforms and artificial intelligence as a basis for enhancing students’ digital skills.” And encouraging the adoption of digital learning processes. At the Arab regional level, ( Al-Aryani, 2024) raised a different analysis in this regard, emphasizing the idea that “implementing digital transformation strategies and improving the quality of education” is conditional on the integration of digital curricula, training of personnel, and the development of a solid physical, service, and digital infrastructure. Student engagement increased by 20-30%. Al-( Alsayed and Al-Assaf, 2023) also found that a lack of strategic planning and inadequate infrastructure were major obstacles to digital transformation at Jordanian universities, while training and other aspects of infrastructure development had a significant impact on the educational process, resulting in a 22% increase in educational efficiency. Study ( Hashim et al., 2022) demonstrated that digital transformation in higher education epresents a roadmap for building a sustainable competitive advantage for universities by supporting strategic management and improving the student experience in light of rapid global changes. More broadly, ( Alzahrani, 2022) conducted a systematic study of the application of artificial intelligence in education in Arab countries. This study identified advantages of this technology, including personalized learning, administrative efficiency, and support for distance learning. However, issues related to infrastructure and training continue to hinder the widespread adoption of these technologies. ( Al-Slehat et al., 2023) also noted that the use of artificial intelligence in higher education requires technological, human, and financial resources, in addition to human factors such as qualified and dedicated staff. Internationally, recent research has demonstrated that AI assistants can effectively provide personalized learning experiences, thereby reducing students’ cognitive burden and improving their academic achievement ( Choi et al., 2024). ( Katsamakas et al., 2024) have shown that investing in AI in higher education can improve learning, research, and management. Research on ChatGPT and other AI technologies prioritizes ethical concerns and emphasizes overreliance on controls to avoid negative impacts on critical thinking and educational quality ( Dos, 2025; Howland, 2025).( Wang et al., 2024) This study demonstrated that AI in education enhances personalized learning and the effectiveness of educational systems despite technical and ethical challenges. This presents important opportunities for future research in the areas of educational justice and social interaction. ( Behera et al., 2025) This study examines fairness, accountability, transparency, and ethics in AI in higher education as concluded by the predominance of descriptive and qualitative definitions, with an emphasis on integrating different approaches in future research.

Based on a wealth of local, regional, and international research on this topic, we identified a lack of research that explores the integration of AI, digital transformation, and digital innovation in higher education through a holistic approach, highlighting the interrelationships between these concepts. This deficiency plagues most existing research, particularly in Iraq. Therefore, the need for a comprehensive study using AI as the sole variable, digital transformation as the mediating variable, and digital innovation as the dependent variable became apparent. This is the goal of our current research. We aim to provide practical, strategic recommendations to support decision-making and improve the quality of digital education in Iraqi universities. This research also aims to inform decisions that improve educational quality.

## 4. Theoretical aspect

### 4.1 Artificial intelligence in higher education

Since the mid-20th century, universities have undergone significant changes due to increased digitization. One of the most significant advances has been the emergence of artificial intelligence (AI). In 1956, John McCarthy’s team at the Dortmund Symposium pioneered the early development of AI, establishing a new science aimed at developing systems that mimic human cognitive abilities ( Norvig and Russell, 2010). Since then, AI has transcended from a branch of computer science to become a powerful tool, boosting performance in diverse fields such as healthcare, transportation, and entertainment. These sectors have benefited from its ability to automate most academic and administrative tasks ( Saxena and Doleck, 2023). As research has advanced, AI has been applied to techniques that mimic human reasoning, such as self-learning, prediction, and decision-making ( Abrokwah and Larbi-Oko, 2024; Zakharov, 2021). Recent research suggests that the effectiveness of these technologies in education depends crucially on whether educational institutions can enhance students’ competence in areas such as digital literacy and data literacy and cultivate the technical skills necessary to effectively use these tools ( Wang, Sun, and Chen, 2023). Research indicates that artificial intelligence (AI) extends beyond machine learning or data processing to encompass artificial neural networks and language processors capable of adapting to new data and predicting outcomes ( Baker and Smith, 2019; Popenici and Kerr, 2017). ( Luckin et al., 2016) categorize AI applications in education into three main areas: intelligent tutoring systems, collaborative tools, and virtual reality technology. These tools help design personalized learning paths for students, enhance interactivity, and provide more diverse learning experiences ( Salmon, 2000; Jonassen et al., 1995). In this context, it can be said that AI constitutes an integral component of higher education systems at three different levels: at the student level, through personalized educational support; at the faculty level, through tools to improve teaching; and at the institutional level, through systems that support management and strategic decision-making ( Joseph, 1999). Thus, we propose the following:

H1: Artificial intelligence has a positive and statistically significant impact on promoting digital innovation at the University of Ninevah.

### 4.2 Digital innovation in education

Digital innovation enables the use of digital technologies in the development of services and products, enhancing institutional integrity and value ( Nambisan et al., 2017). The concept of digital innovation is not merely an expansion of traditional innovation; rather, it represents a qualitative shift in the creation of new, more innovative, and creative practical solutions. These solutions rely on digital platforms and smart application practices that facilitate collaboration between all stakeholders and enable them to access and enter markets in innovative, unconventional ways. The importance of digital innovation is also evident in the integration of physical and virtual systems, resulting in hybrid products and services that combine the physical and the digital, such as smart devices and interactive applications. However, these transformations have a significant impact on traditional leadership, necessitating a leadership style capable of handling the complexity and rapid developments resulting from digital transformations ( Svahn et al., 2017). In Arab and Iraqi societies, digital innovation is still in its infancy and faces obstacles related to a lack of infrastructure and a lack of digital literacy among academic staff. This necessitates the development of long-term plans that create a supportive environment for innovation and integrate human, technical, and organizational resources ( Ahmed et al., 2022). It is recognized that the success of digital innovation depends on the ability of educational institutions to establish sustainable and appropriate governance frameworks for the educational process, with a focus on improving the quality of their academic outcomes, expanding the scope of blended learning, and developing digital curricula that align with global trends ( El Said, 2021). Thus, we propose the following:

H2: Artificial intelligence has a positive and statistically significant impact on supporting digital transformation at the University of Ninevah.

### 4.3 Digital transformation in education

**Figure 1.**
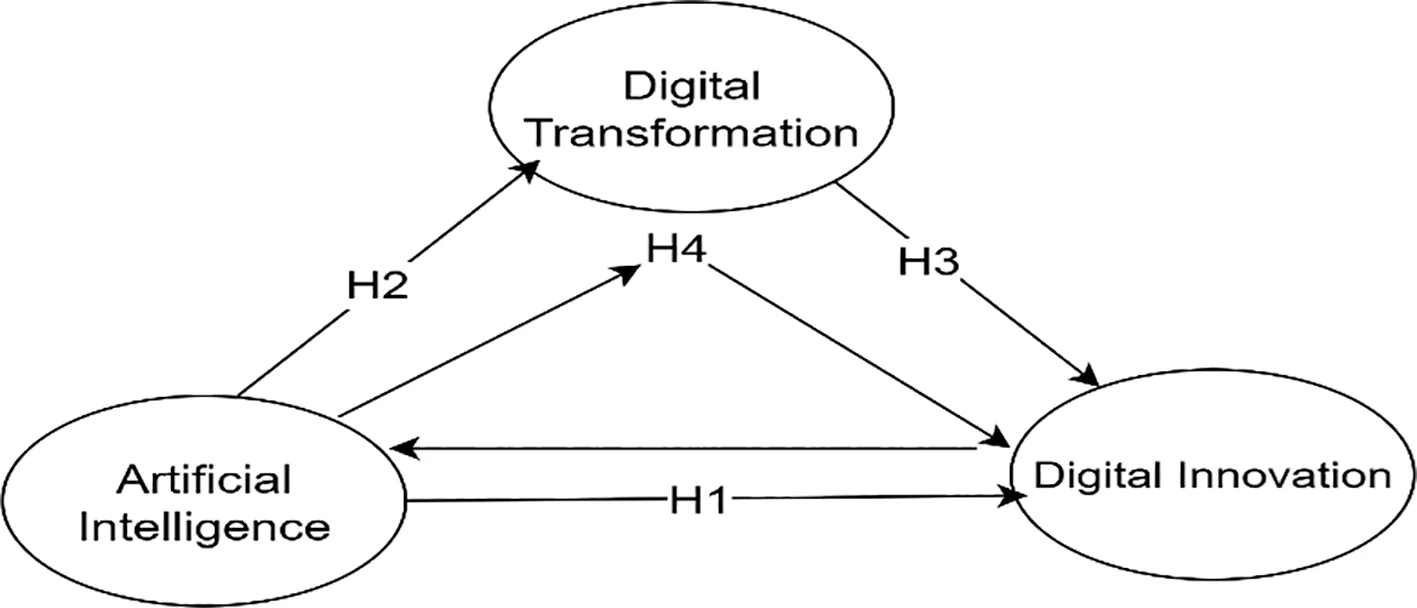
Hypothetical relationships.

Digital transformation plays a significant role in integrating digital technology into various aspects of business, including the education sector. The goal is to improve customer service and enhance operational efficiency. Furthermore, it includes redesigning a company’s or organization’s structure, changing its culture, and developing its leadership style to keep pace with new changes ( Westerman et al., 2014). ( Liu et al., 2023) explained that digital transformation cannot be limited to developing technological capabilities, but can be expanded to include moving from the traditional approach to an integrated business model based on digital technology to create new products and services. Digital transformation encourages the creation of new revenue streams by enhancing the value of services provided to the public, and education services are certainly one of these. Meanwhile, ( Vial, 2019) noted that “digital transformation is a complex process that requires continuous interaction between technological resources and organizational capabilities,” leading to significant and clear changes in the business structure ( Matt et al., 2015). Rather, it depends primarily on the ability of administrators and leaders to build a distinct digital culture within the organization and educate employees on the importance of adapting to the requirements of the digital age. This also applies to educational institutions such as universities and other institutions operating in this field. Digital transformation has become an important and necessary aspect of improving the quality of education and academic knowledge management. Universities that have implemented digital transformation and pursued a strategy of adopting digital technology have been able to establish a broad base for an educational system based on e-learning and have acquired significant capacity to handle big data. It has also been able to provide more comprehensive, flexible, and modern educational services, enhancing its capacity for innovation in various forms and across diverse disciplines, and ensuring the sustainability of its institutional performance over the long term ( Benavides et al., 2020). Study ( Hess et al., 2020) clarified the differences between “digital transformation” and simple “digitization.” Simple digitization refers to the conversion of paper data into digital form, while “digital transformation” refers to a strategic shift aimed at creating new value, under clear and advanced leadership with effective management capable of absorbing, leveraging, and developing change to achieve comprehensive, continuous, and sustainable development ( Reis et al., 2018). ( Verhoef et al., 2021) explained that digital transformation is now considered a prerequisite for organizational success, rather than a secondary option, as it contributes to improving customer experience, increasing organizational agility, and enhancing the ability to innovate. ( George and Paul, 2020) also view digital transformation as a complex process that relies on the continuous interaction between modern technology, human resources, and the organization. Practical experience shows that organizations that implemented integrated digital transformation strategies were better able to deal with crises, such as the COVID-19 pandemic, which accelerated digital transformation across various sectors, especially education Figure 1 shows the proposed relationships in this study, Thus, we propose the following:

H3: Digital transformation has a positive and statistically significant impact on promoting digital innovation at the University of Ninevah.

## 5. Method

This research approach combines descriptive and quantitative analysis, using statistical tools, tables and charts, and a five-point Likert-scale questionnaire. The questionnaire was administered exclusively to faculty members at the University of Ninevah and was evaluated by experts in the field of artificial intelligence and digital transformation to ensure its validity ( Hair et al., 2019; Creswell, 2014).

### 5.1 Participants

Participants received an email explaining the study and inviting them to participate. Those who agreed clicked a link in the consent letter attached to the invitation, which redirected them to the survey. The email invitation was received by 581 faculty members currently working at the university, and a total of 206 faculty members responded (35% response rate). The sample comprised faculty members representing various academic and research ranks from various departments and universities, including faculty members working in residence at the university.

**Ethical approval**

This study was conducted at the University of Ninevah in Iraq. According to the institutional practices at the time of data collection, no formal ethics committee review process or approval number was available for studies involving academic staff in non-invasive survey-based research. However, the study adhered to the principles of the Declaration of Helsinki, and participation was voluntary, anonymous, and confidential.

**Informed consent**

Informed consent was obtained verbally from all participating university faculty members. Verbal consent was chosen because written consent is not required in non-invasive survey studies conducted within Iraqi universities, and because participants preferred fast and anonymous participation. All participants were informed about the purpose of the study, confidentiality of responses, and their right to withdraw at any time.

### 5.2 Demographic characteristics

The demographic characteristics of the study sample are presented in Table 1, which shows that 55.8% (n = 115) of the respondents were men (compared to n = 91 women). The results also indicate that 62.1% of the respondents were teaching assistants, lecturers 26.6% were assistant professors, and only 9.4% were full professors 1.9%. The number of respondents under the age of 30 was 12, equivalent to 5.8% of the sample group of 206. The total number of respondents aged 30-39 was 147, equivalent to 71.4%, while those aged 40-49 were 29, equivalent to 14.1%. Those aged 50-59 were 17, equivalent to 8.2%, while those aged 60 and over were 1, equivalent to 0.4%. All university faculties participated in the survey, with 22.1% of respondents employed at the Faculty of Engineering, 16.5% at the Faculty of Information Technology, 17.5% at the Faculty of Law, 12.1% at the Faculty of Medicine, and 12.6% at the Faculty of Nursing (9.2%), the Faculty of Pharmacy (17%), and the University Presidium (15%).

**Table 1.** Sample demographic characteristics.

Category	Frequency	Percent
Gender		
Male	115	55.8
Female	91	43.7
Age		
Less than 30 years	12	5.8
30 to 39 Years	147	71.4
40 to 49 Years	29	14.1
50 to 59 Years	17	8.2
60 Years and older	1	0.4
Academic Title		
Professor	4	1.9
Assistant Professor	19	9.4
Lecturer	53	26.6
Lecturer Assistant	124	62.1
College		
University Presidency	31	15
College of Medicine	26	12.6
College of Pharmacy	35	17
College of Engineering	34	16.5
College of Nursing	19	9.2
College of Information Tech	36	17.5
Faculty of Law	25	12.1

### 5.3 Measures

All scales are composite. Each scale is a previously published and validated composite scale. Unless otherwise specified, the means of the items in each scale were:

Each scale is a composite measure, each of which has been previously published and validated unless otherwise specified. The means of the items in each scale were: Perceptions of Artificial Intelligence (AI), which was measured as an independent variable, composed of three dimensions: general perceptions of AI, current use of AI, and future impact of AI. Each of the three dimensions above consists of four items measured using a five-point (Likert) scale (1 = strongly disagree, 5 = strongly agree). Examples of items include “AI contributes to improving the quality of higher education,” “AI enhances interaction between students and faculty members,” and “I benefit from smart learning platforms that rely on AI to evaluate my academic performance.” The Cronbach’s alpha coefficient for this scale was 7.46, indicating good internal consistency. Digital transformation (DT) was also measured as an intermediate variable (M) consisting of three dimensions, each of which consists of four items: the institution’s digital strategy, the implementation of digital transformation in the learning process, and the university’s readiness for digital transformation. Examples of this are: “The university provides the necessary infrastructure for digital transformation,” “Digital transformation helps in monitoring academic progress better,” and “Universities that implement digital transformation achieve better performance.” A five-point Likert scale was used (1 = strongly disagree, 5 = strongly agree). The Cronbach’s alpha coefficient for this scale was (0.773), indicating good internal consistency. Digital innovation as a dependent variable (DI) consists of three dimensions: (technological development in education, the use of digital innovation in the learning process, and the future impact of digital innovation). Each dimension consists of four items. Examples of this include: “The university encourages digital innovation within its learning strategy,” “Provides technical support to users of digital systems,” and “The university provides the infrastructure.” Necessary for digital transformation. A five-point Likert scale was used (1 = strongly disagree, 5 = strongly agree). Cronbach’s alpha coefficient was (.797), indicating very good internal consistency.

### 5.4 Analysis

In our current research, we used structural equation modeling (SEM) using AMOS software. For our hypotheses, we presented four main hypotheses: one explains the direct influence relationship between AI and digital innovation, and the other the indirect influence relationship between AI and digital innovation with the mediating variable, digital transformation. Before delving into the details of testing these hypotheses, it is necessary to properly test the significance of the hypothetical model and present it in its general form. It is necessary to test the relationships between the independent and dependent variables independently, between the independent and mediating variables, and between the mediating and dependent variables. We must ensure that these tests include significant influence relationships so that the hypothetical model can be comprehensively tested as a single package. Table 2 also presents the descriptive statistics, reliability coefficients, and correlations.

**Table 2.** Study variable means, standard deviations, and correlations.

Variable	M	SD	AI	DI	DT
1. Gender	1.41	.494			
2. Age	2.32	.780			
3. Academic Title	3.49	.668			
4. College	3.76	2.03			
5. Artificial intelligence	3.47	.751	1		
6. Digital innovation	2.95	.573	.325*	1	
7. Digital transformation	3.64	.794	.603*	.469*	1

Notes: (*) significant at %1, (**) significant at %5, (***) significant at %10.

## 6. Results

Therefore, we will proceed through the tests according to the following paragraphs:

### H1: Artificial intelligence has a positive and statistically significant impact on enhancing digital innovation at the University of Ninevah

**Figure 2.**
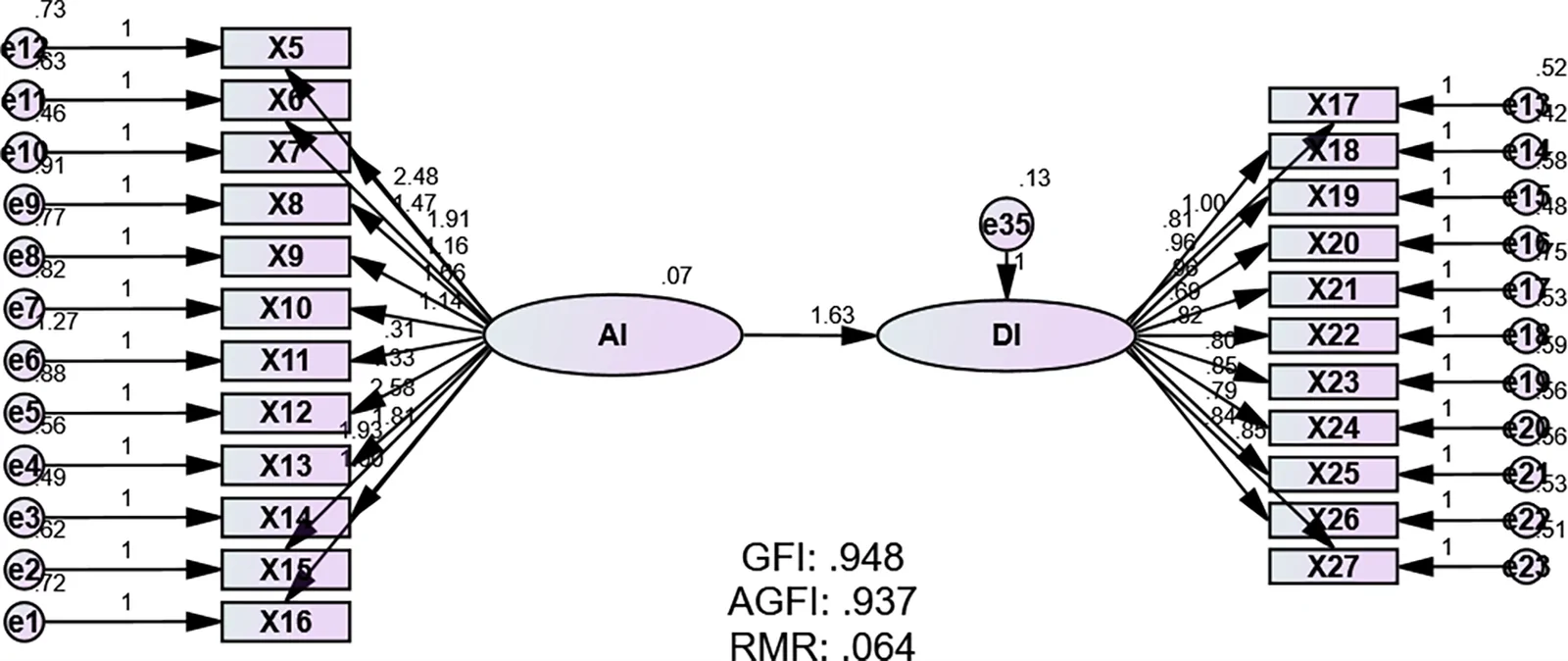
Confirmatory factor analysis of the variables and dimensions of artificial intelligence, digital innovation, and digital transformation.

Figure 2 illustrates the direct positive impact of artificial intelligence on digital innovation at a statistical significance level of 0.05. This hypothesis was met, as evidenced by the fit values that were within acceptable levels. The Goodness of Fit (GFI) value was 0.948, and the AGFI (Adjusted Goodness of Fit) value was 0.937, which is above the acceptable levels (above 90%). The Root Mean Square Residual (RMSR) also reached 0.064, which is lower than the standard rate for model acceptance. According to ( Bentler, 1990), the RMR should not exceed 0.080, indicating the quality of the direct impact of artificial intelligence on digital innovation. This enables us to accept the first hypothesis, which confirms the existence of this direct impact. Figure 2, the direct positive impact of artificial intelligence on digital innovation. Table 3 shows the impact values of artificial intelligence on digital innovation in detail. The regression values were positive, amounting to 1.630, which is a direct, positive impact relationship, meaning that the more the use of artificial intelligence tools increases, the more digital innovation will increase, which confirms this positive impact. The confidence level values were also positive, indicating the significance of the impact, which enables us to accept the first hypothesis. The significance of the P value was less than 0.05, indicating the acceptance of the first hypothesis.

**Table 3.** Indicators of the direct impact of artificial intelligence on digital innovation.

Independent variable	Relationship direction	Dependent variable	Impact value	Confidence Interval 95%		P- value
				Lower	Upper	
(AI)	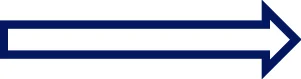	(DI)	1.630	0.999	3.920	0.004

### H2: Artificial intelligence has a positive and statistically significant impact on supporting digital transformation at the University of Ninevah

**Figure 3.**
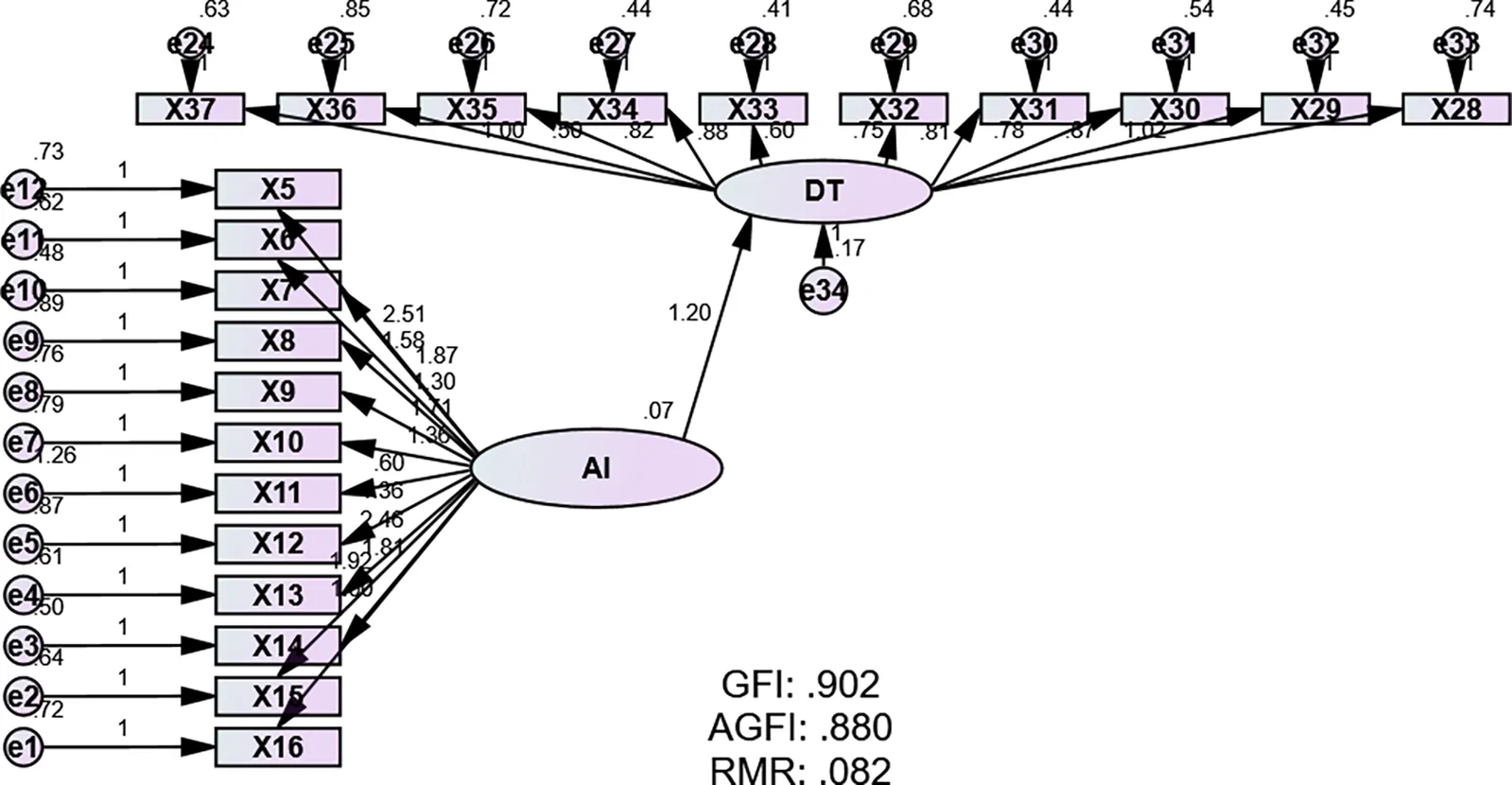
The direct impact of artificial intelligence on the mediating variable of digital transformation.

One of the requirements for proving the relationships in the hypothetical diagram is the second relationship between the independent variable (AI) and the mediating variable (digital transformation). This must have a direct, positive, and unidirectional impact. This was achieved through Figure 3, which illustrates the impact of artificial intelligence on digital transformation, as measured by the matching values, which were within acceptable levels. The GFI (Goodness of Fit) value was 0.902, and the AGFI (Adjusted Goodness of Fit) value was 0.880, which are within acceptable levels (above 90%, except for the AGF value). The RMR index was slightly higher than the acceptable level of 0.082, which is greater than 0.080. This does not represent a strong impact on the quality of the model, given that the remaining indicators are within acceptable levels, indicating the quality of the direct impact of artificial intelligence on digital transformation. To test the second hypothesis in detail, we note the positive impact relationship between artificial intelligence and direct digital transformation, as indicated by the impact value of 1.204, and at the positive confidence level within the highest level of 2.334 and the lowest level of 0.782, both levels are positive, indicating the significance of the model as shown in Table 4. Through the significance value of P, we can accept the second hypothesis, which states that there is a significant impact of artificial intelligence on the mediating variable, direct digital transformation. This is a second proof of the acceptance of the hypothetical model and the significance of the impact between the independent and dependent variables.

**Table 4.** Indicators of the direct impact of artificial intelligence on the mediating variable digital transformation.

Independent variable	Relationship direction	Dependent variable	Impact value	Confidence Interval 95%		P- value
				Lower	Upper	
(AI)	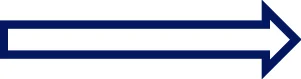	(DT)	1.204	0.782	2.334	0.006

### H3: Digital transformation has a positive and statistically significant impact on enhancing digital innovation at the University of Ninevah

**Figure 4.**
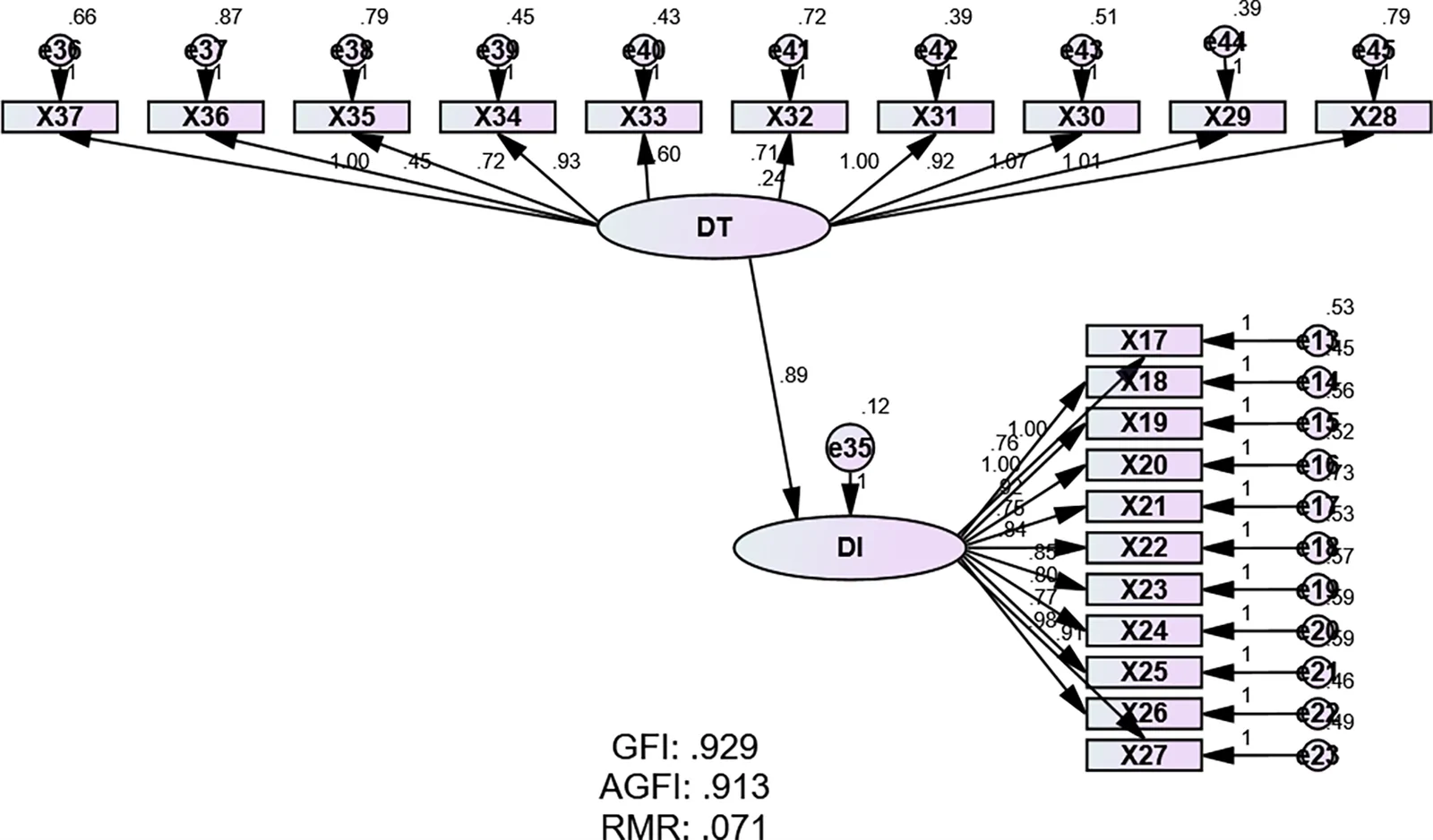
The direct effect of the mediating variable, digital transformation, on digital innovation.

Figure 4 shows the matching values between the mediating variable (digital transformation) and the dependent variable (digital innovation). The impact relationship was positively proportional; the greater the digital transformation, the greater the digital innovation, as indicated by the impact value of 0.892 in Table 5. The Root Mean Square Residual (RMSR) was 0.071, which is lower than the standard rate for model acceptance and should not exceed 0.080. The GFI and AGFI values reached 0.92 and 91%, respectively, indicating the quality of the hypothetical scheme in terms of the impact of the mediating variable (digital transformation) on digital innovation. This indicates the quality of the hypothetical model in terms of the impact of the mediating variable (digital transformation) on digital innovation. The greater an educational organization's ability to utilize, develop, and improve technological infrastructure in its operations, the greater the digital transformation of those operations at both the operational and strategic levels. Digital transformation adds a crucial element to information quality: speed and accuracy in organizing information for the educational organization. Consequently, flexibility in delivering educational services and the transition to the digital level become easier due to the programming, standardization, and coding of all service-related elements. Regarding the indicators of testing the H3, we note that it has been achieved at the level of the current research. Through the value of the positive and direct effect of 0.892, we note that the third hypothesis (the impact of digital transformation on digital innovation) has been achieved. The confidence levels were significant, as they were in the correct direction, which is a one-way positive direction indicating the significance of the effect. The highest level of confidence reached 1.458 and the lowest 0.566, both of which were in one direction positive indicating the significance of the effect. This is confirmed by the p value, which was significant and reached 0.006, which enables us to accept the third hypothesis, which states: “There is a significant impact of digital transformation on digital innovation at the significance level for human research, which is 0.05”.

**Table 5.** Indicators of the impact of the mediating variable (digital transformation) on the dependent variable (digital innovation).

Mediating variable	Relationship direction	Dependent variable	Impact value	Confidence Interval 95%		P- value
				Lower	Upper	
(DT)	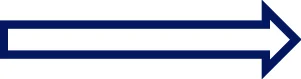	(DI)	0.892	0.566	1.458	0.006

### H4: The impact of artificial intelligence on digital innovation, with the presence of the mediating variable as a basic condition in this relationship, at a significance level of 0.05

**Figure 5.**
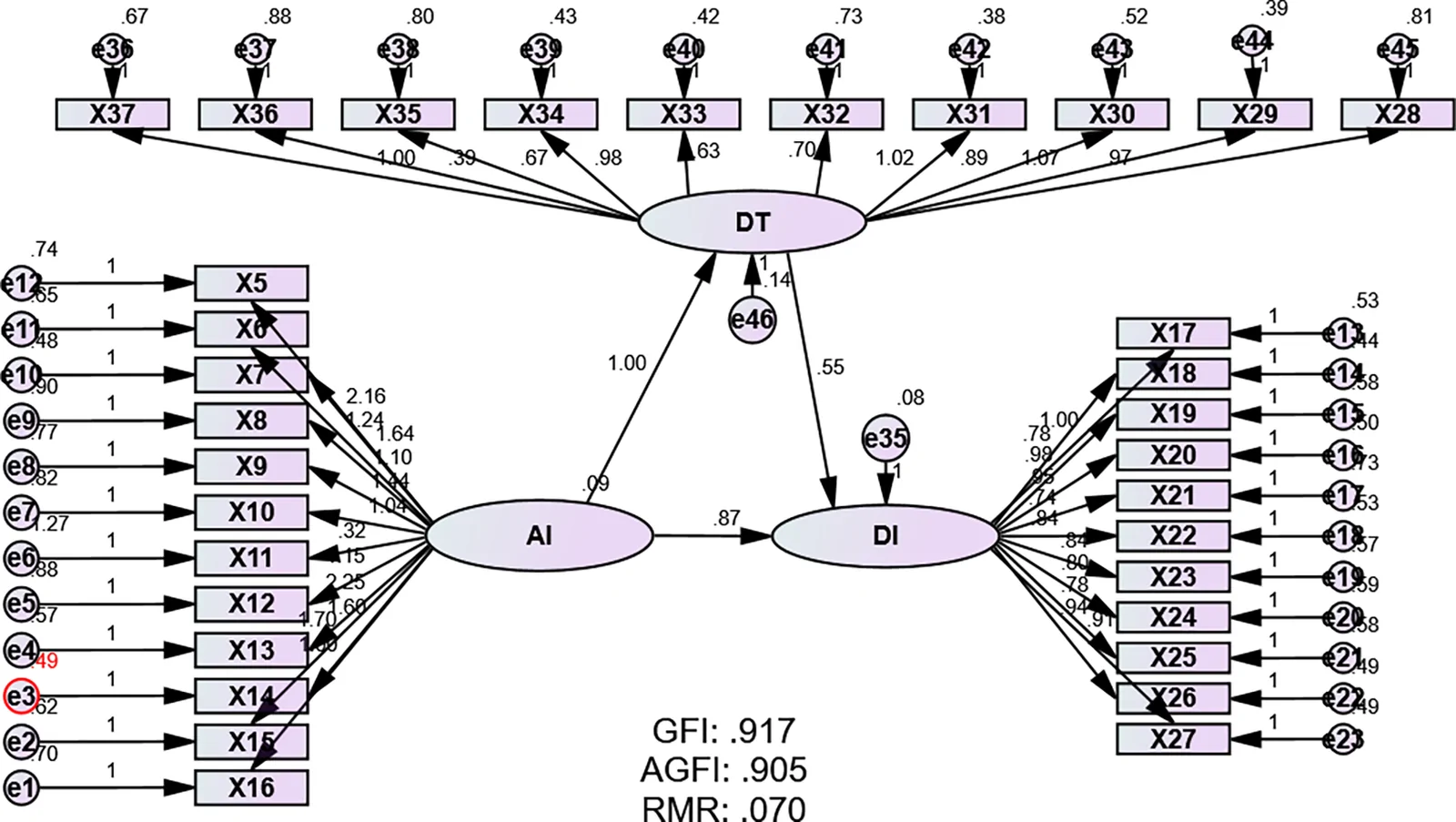
The relationship between the direct and indirect impact of the mediating variable (The impact of artificial intelligence on digital innovation in the presence of the mediating variable digital transformation).

After testing the components of the hypothetical scheme presented by the current research, we can now, through Table 6 and Figure 5, test the fourth hypothesis, which expresses the research objective, problem, and basic idea: “Does artificial intelligence impact digital innovation, with the presence of the mediating variable, namely digital transformation?” Figure 5 shows that we can present the hypothetical diagram as a single package and it can be used after a long period of time so that it gives the same results because we tested its parts and the influence relationships it presented and they were all significant and with acceptable confidence levels. From Figure 5, it is clear that the value of the square root of the mean of the residuals (RMR) was within the acceptable rate, which amounted to 0.070, indicating the model’s ability to represent the influence relationship between the variables. The model is also characterized by quality through the GFI and AGFI values, which amounted to 0.917 and 0.905, respectively, indicating the quality of the model and the absence of significant differences between the hypothetical model presented by the current research in the methodology and the applied model on which the opinions of the respondents in the researched organization were surveyed. Table 6 shows the detailed impact relationship and the degree of significance of the direct and indirect impact, and whether the role of the mediating variable has a complete impact, a partial impact, or no impact of the mediating variable. From Table 6, we note that the value of the direct impact was 0.865, which represents a direct relationship, i.e., the more the use of artificial intelligence increases, the more digital innovation increases. This is a logical relationship, as artificial intelligence provides many solutions in various academic and research fields. The confidence levels at the 95% level were at the lowest level of 0.402 and the highest level of 1.766, which is positive in one direction, indicating the significance of the direct impact, as indicated by the p-value, which reached 0.005, which is less than the standard significance level in human studies, which is 0.05. The essence of the model and the title of the current research can be tested in the relationship between the independent variable, artificial intelligence, and the dependent variable, digital innovation, in the presence of the mediating variable, digital transformation. This was achieved through the data in Table 6, as the value of the positive indirect effect reached 0.554, which is a positive effect that explains a logical relationship despite the entry of the mediating variable. In other words, there is an effect of artificial intelligence on digital innovation, but on the condition of the presence of digital transformation. The more artificial intelligence and digital transformation increase, the more digital innovation increases in the organization under study. This value also indicates that the mediating variable has a partial effect, as the effect is distributed between the independent variable and the mediating variable to affect together the dependent variable, digital innovation, as indicated by the lowest level of 1.208 and the highest level of 2.71 of the confidence levels and as indicated by the P value of 0.002, which is greater than the standard level in human studies of 0.05. This also indicates the significance of the indirect effect, in other words, the distribution of the effect between the independent variable and the mediating variable in the dependent variable. In other words, in order to achieve digital innovation, the effect must be distributed between artificial intelligence and digital transformation in order for that innovation to be achieved, which enables us to accept the fourth hypothesis, which states, “There is an effect of artificial intelligence on digital innovation with the presence of digital innovation in the educational organization at a significance level greater than or equal to 0.05”.

**Table 6.** The relationship between the direct and indirect influence of the mediating variable (the impact of artificial intelligence on digital innovation in the presence of the mediating variable digital transformation).

Independent variable	Relationship direction	Dependent variable	Impact value	Confidence Interval 95%		P- value
				Lower	Upper	
(AI)	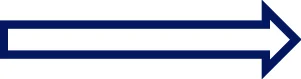	DI	0.865	1.766	0.402	0.005
(AI)	Through (DT)	DI	0.554	2.71	1.208	0.002

## 7. Discussion

This study aims to measure the impact of artificial intelligence (AI) on the digital transformation of faculty members and digital transformation at the University of Ninevah, both directly and indirectly. The results presented provide strong support for the proposed hypotheses, contributing to fundamental practical and theoretical contributions to the study of AI in university settings. We find strong support for the first hypothesis (H1), which indicates that AI directly improves the process of digital innovation. Faculty members’ opinions are more inclined to use AI to improve the quality of education. This study is consistent with previous studies showing that AI improves the quality of higher education ( Ren & Wu, 2025; Ofosu, 2024). The relationship between artificial intelligence and digital transformation was investigated in (H2), which focused on the role of digital transformation as a mediator. The results showed a direct and statistically significant effect of artificial intelligence on digital transformation. This indicates that faculty members’ interest in digital transformation increases efficiency and provides better service through artificial intelligence. This is consistent with previous studies, which confirm that digital transformation increases effectiveness and better participation ( Fernández et al., 2023; Molina-Carmona and García-Peñalvo, 2025). The relationship between artificial intelligence, digital innovation, and digital transformation was also examined as a mediator in the third refusal. The results showed a statistically significant indirect effect. This indicates that digital transformation indirectly influences artificial intelligence and digital innovation, as faculty members believe that the presence of digital transformation will facilitate the application of artificial intelligence technologies and other digital innovation technologies. In the field of higher education, this is consistent with the study ( Orlando et al., 2025). The H4, explores the relationship between the three variables, both directly and indirectly. The results showed that there is a direct and indirect statistically significant effect on the variables. This indicates that digital transformation, as a mediator, indirectly affects each variable. The results indicate that the use of digital transformation in universities as a means of using artificial intelligence to improve the quality of education and motivate faculty members to actively participate in digital innovations, has positive effects on the educational level in Iraqi universities.

## 8. Limitations

This study provides important insights into the impact of artificial intelligence on digital innovation and digital transformation. However, There are some limitations that must be taken into account. These include that our study was conducted within a limited geographical context, namely the University of Ninevah, as part of Iraqi higher education. This limits the generalizability of the results to other universities. A self-administered questionnaire was also used to collect data, the 280 individuals only 206 responses were received. This indicates a disparity and variation in the level of support provided by staff in educational institutions for research activities which in turn leads to some bias in responses due to social factors among participants, although we directed that responses be honest and transparent. Our study relied on a cross-sectional design, meaning that it was conducted at a single point in time, which may not allow for the establishment of direct causal relationships between variables.

## 9. Future research

Based on the above-mentioned limitations, the study recommends that researchers increase the samples more widely within the scope of Iraqi universities, including private universities, to spread and improve the benefit. In addition, artificial intelligence should be applied in universities and attention should be paid to digital transformation and digital innovation, as they have an effective role in improving the quality of education and increasing the efficiency of the performance of educational and administrative institutions in general and the teaching staff in particular. The study also recommends that researchers use various research methodologies, such as longitudinal or experimental studies, to test the causal relationships between artificial intelligence and other variables.

## 10. Conclusion

Artificial Intelligence (AI) and digital innovation do positively influence the quality of education and administrative efficacy in Iraqi universities, enabled largely by digital transformation. Improved teaching methodology, elevated student engagement, and boosted research capabilities are fruits enjoyed by universities that have embraced digital transformation. Infrastructure inadequacy and other challenges such as the gap in digital literacy and resistance to change hinder smooth implementation though investment in digital transformation is a priority at this stage to fully obtain the benefits of (AI) and digital innovation (DI) in Iraqi higher education.

## Data Availability

Repository name: The Impact of Artificial Intelligence in Enabling Digital Innovation/The Mediating Role of Digital Transformation: A Survey Study Opinions of a Sample for Faculty Members at University of Ninevah. https://doi.org/10.5281/zenodo.17754880. MOHAMMED, T. M., Raghad, O. J., MOATASEM, H. M. S., Adel, M. A., Nizar, S. alqahwachi., & WSAM, H. F. (2025). The project contains the following underlying data:
- Dataset.spss (De-identified participant responses, including variables such as gender age, Academic Title, Workplace and Likert-scale responses). Repository name: The Impact of Artificial Intelligence in Enabling Digital Innovation/The Mediating Role of Digital Transformation: A Survey Study Opinions of a Sample for Faculty Members at University of Ninevah. https://doi.org/10.5281/zenodo.17754880. MOHAMMED, T. M., Raghad, O. J., MOATASEM, H. M. S., Adel, M. A., Nizar, S. alqahwachi., & WSAM, H. F. (2025). This project contains the following extended data:
- Questionnaire.PDF (The survey questionnaire used in this study). - Data are available under the terms of the Creative Commons Zero “No rights reserved data waiver” (CC0 1.0 Public domain dedication). **Note on sensitive data**: All datasets have been de-identified and do not contain any information that could identify participants. Therefore, all data are publicly available in the repository Zenodo.
