# Supplementary material for: The Impact of Artificial Intelligence in Enabling Digital Innovation/ The Mediating Role of Digital Transformation: A Survey Study Opinions of a Sample for Faculty Members at University of Ninevah
Source: F1000Res. 2026 Jul 23;15:312. Originally published 2026 Feb 24. [Version 3] doi: 10.12688/f1000research.173517.3 (PMC13554735; doi:10.12688/f1000research.173517.3)
Supplement: Peer review history

# Reviewer report: Reviewer response for version 3

**DOI:** 10.5256/f1000research.206596.r504098

**Published:** 2026-07-31

**Authors:** Laura Trujillo Liñán

**Affiliations:** 1 Universidad Panamericana, CDMX, Mexico

I would like to congratulate the authors on this third version of the manuscript. Unlike the previous round, the revisions here are substantive and made in the manuscript itself, and they respond directly to the concerns raised in my earlier reports.

 All of the reporting errors identified in version 2 have been corrected: the Cronbach's alpha for the AI scale now reads 0.746; the sample size is now internally consistent (581 invited, 206 respondents) across Section 5.1 and the Limitations section; the 95% confidence intervals in Table 6 have been properly ordered and the interpretation of the p-value under H4 is now correct; and the demographic reporting in Section 5.2 and Table 1 has been rewritten so that frequencies and percentages are coherent, with Table 2 now including standard notation.

 More importantly, the conceptual concerns have been meaningfully addressed. The Discussion now explicitly acknowledges that organizational culture, leadership support, governance structures, financial resources, and policy environments are important factors alongside AI capability, and warns against inferring that AI is the sole enabler of institutional change — precisely the corrective to a technologically deterministic reading that I had requested. The Future Research section has been substantially expanded to recommend the inclusion of multiple stakeholder groups (students, administrators, policymakers, and IT personnel), mixed methods and longitudinal designs, and the incorporation of contextual variables. These additions position the study honestly as a context-specific, exploratory contribution, which is what its design supports.

 A few minor editorial corrections remain, which I trust the authors can address without difficulty and which do not affect my overall assessment:

1. The heading of H4 now reads "The impact of artificial intelligence on digital transformation, with the presence of the mediating variable" — since digital transformation is itself the mediator, this should read "digital innovation" (the heading was correct in version 2).
2. The closing statement of H4 still reads "with the presence of digital innovation" where "digital transformation" is meant, and "at a significance level greater than or equal to 0.05" should be "less than or equal to 0.05."
3. In the Discussion, "the third refusal" should read "the third hypothesis," and the opening sentence repeats "digital transformation" where "digital innovation" appears to be intended.
4. The notes to Table 2 now combine the earlier convention "( *) significant at %1, ( **) %5, (***) %10" with the new line "p < 0.05 (two-tailed)"; the authors should retain only one, correctly formatted convention.
5. For full consistency, it would also be desirable for the Conclusion to reflect the more nuanced framing now present in the Discussion, and for the socio-technical perspective announced in the revision note to be anchored in the theoretical section with appropriate references in a future version of this line of research.

In sum, the authors have engaged seriously and constructively with the review process across three versions, and the manuscript is now considerably more rigorous and transparent than at first submission. I am pleased to approve it.

Is the work clearly and accurately presented and does it cite the current literature?

Yes

If applicable, is the statistical analysis and its interpretation appropriate?

Yes

Are all the source data underlying the results available to ensure full reproducibility?

Yes

Is the study design appropriate and is the work technically sound?

Partly

Are the conclusions drawn adequately supported by the results?

Partly

Are sufficient details of methods and analysis provided to allow replication by others?

Yes

Reviewer Expertise:

Philosophy of Technology, IA, Media, Ethics, Metaphysics.

I confirm that I have read this submission and believe that I have an appropriate level of expertise to confirm that it is of an acceptable scientific standard.

# Reviewer report: Reviewer response for version 2

**DOI:** 10.5256/f1000research.205382.r499527

**Published:** 2026-07-16

**Authors:** Laura Trujillo Liñán

**Affiliations:** 1 Universidad Panamericana, CDMX, Mexico

The authors have made a genuine effort to respond to the first round of review, and version 2 shows measurable improvements: the Limitations section now explicitly acknowledges the single-institution scope, the potential biases of a self-administered questionnaire, and the inability of a cross-sectional design to establish causal relationships. The research gap has been reframed to justify the context-specific focus on Iraqi higher education, and the Future Research section incorporates the recommendation to expand samples and adopt longitudinal or experimental designs. I appreciate the authors' detailed response letter.

 However, several of the substantive concerns raised in my first report remain unaddressed in the manuscript itself:

1. The theoretical framing is unchanged. The paper still does not engage with critical perspectives on AI, philosophical or ethical frameworks, or socio-technical systems theory, and the three-variable model continues to omit organizational culture, leadership and governance, economic constraints, and policy environments. Acknowledging model parsimony in a response letter is not equivalent to addressing the resulting technologically deterministic framing, which persists in the Discussion and Conclusion (e.g., the claim that digital transformation leads to better teaching practices and more engaged students).
2. The stakeholder scope remains limited to faculty members. My recommendation to incorporate multiple stakeholders and mixed methods is not reflected in the revised Future Research section.

In addition, version 2 contains reporting errors that must be corrected before the paper can be considered scientifically sound:

1. The Cronbach's alpha for the AI scale is reported as 7.46, an impossible value (presumably 0.746).
2. The sample size is internally inconsistent: Section 5.1 reports 581 invited faculty with 206 respondents (35%), while the Limitations section refers to 280 individuals.
3. In Table 6, the 95% confidence intervals are inverted (lower bound 1.766 vs. upper bound 0.402; lower 2.71 vs. upper 1.208). Relatedly, the text under H4 states that a p-value of 0.002 "is greater than the standard level of 0.05," which is incorrect, and the hypothesis statement refers to "the presence of digital innovation" where "digital transformation" is meant.
4. The demographic reporting (Section 5.2 and Table 1) contains mismatched percentages and garbled phrasing regarding academic titles and college affiliations, and the significance conventions in the notes to Table 2 are non-standard.

So, the authors have improved the transparency of the study's limitations, but the conceptual concerns are answered rhetorically rather than through revision, and the statistical reporting errors listed above compromise the reliability of the presented results as they stand.

Is the work clearly and accurately presented and does it cite the current literature?

Yes

If applicable, is the statistical analysis and its interpretation appropriate?

Yes

Are all the source data underlying the results available to ensure full reproducibility?

Yes

Is the study design appropriate and is the work technically sound?

Partly

Are the conclusions drawn adequately supported by the results?

Partly

Are sufficient details of methods and analysis provided to allow replication by others?

Yes

Reviewer Expertise:

Philosophy of Technology, IA, Media, Ethics, Metaphysics.

I confirm that I have read this submission and believe that I have an appropriate level of expertise to confirm that it is of an acceptable scientific standard, however I have significant reservations, as outlined above.

# Reviewer report: Reviewer response for version 2

**DOI:** 10.5256/f1000research.205382.r499526

**Published:** 2026-07-13

**Authors:** Abeer Alkhwaldi

**Affiliations:** 1 Mutah University, Karak, Jordan

The authors addressed all the required comments satisfactorily.

Is the work clearly and accurately presented and does it cite the current literature?

Yes

If applicable, is the statistical analysis and its interpretation appropriate?

Yes

Are all the source data underlying the results available to ensure full reproducibility?

No source data required

Is the study design appropriate and is the work technically sound?

Yes

Are the conclusions drawn adequately supported by the results?

Yes

Are sufficient details of methods and analysis provided to allow replication by others?

No

Reviewer Expertise:

NA

I confirm that I have read this submission and believe that I have an appropriate level of expertise to confirm that it is of an acceptable scientific standard.

# Reviewer report: Reviewer response for version 1

**DOI:** 10.5256/f1000research.191344.r482042

**Published:** 2026-06-30

**Authors:** Mohammed Jaboob

**Affiliations:** 1 Dhofar University, Salalah, Oman

The paper is scientifically sound in its current form and only minor, if any, improvements are suggested.

 - Clarify the research gap more explicitly , why is the Iraqi higher education context unique compared to global studies?

 - Add a brief note on limitations (e.g., data availability, institutional readiness) to make the study more academically rigorous.

Is the work clearly and accurately presented and does it cite the current literature?

Yes

If applicable, is the statistical analysis and its interpretation appropriate?

Yes

Are all the source data underlying the results available to ensure full reproducibility?

Yes

Is the study design appropriate and is the work technically sound?

Yes

Are the conclusions drawn adequately supported by the results?

Yes

Are sufficient details of methods and analysis provided to allow replication by others?

Yes

Reviewer Expertise:

NA

I confirm that I have read this submission and believe that I have an appropriate level of expertise to confirm that it is of an acceptable scientific standard.

## References

[1] : Integration of Generative AI Techniques and Applications in Student Behavior and Cognitive Achievement in Arab Higher Education. *International Journal of Human–Computer Interaction* .2025;41(1) : 10.1080/10447318.2023.2300016 353-366 10.1080/10447318.2023.2300016

# Reviewer report: Reviewer response for version 1

**DOI:** 10.5256/f1000research.191344.r482040

**Published:** 2026-05-23

**Authors:** Abeer Alkhwaldi

**Affiliations:** 1 Mutah University, Karak, Jordan

**Minor Comment 1: Literature Review – AI for Education Citations**

**Section:** 3. Literature Review (Pages 4–5)

**Comment:** The international subsection of the literature review mentions personalized learning and the general benefits of AI assistants, but it could be updated with more recent and comprehensive foundational studies regarding AI’s implementation in education. To provide a stronger academic context for higher education institutions, please integrate and cite the following papers:

- **Papers that are recommended to be cited:**
- Reference 1

- Reference 2

**Minor Comment 2: Variable Labeling Consistency**

**Section:** 2. Research Model & 4.3 Digital Transformation (Pages 4 & 6) **Comment:** There are minor typographical discrepancies in the abbreviations used for the variables. In Section 2, digital transformation is abbreviated as " **TD**" (e.g., *"directly affects digital innovation (DI) and digital transformation (TD)"*) , whereas in Section 5.3 and throughout the analysis sections, it is correctly referred to as " **DT**". Please review the manuscript and standardize this abbreviation to " **DT**" across all sections to prevent confusion for the reader.

**Minor Comment 3: Clarification of "Digitization" vs "Digital Transformation"**

**Section:** 4.3 Digital Transformation in Education (Page 6) **Comment:** In paragraph 5 of this section, the text correctly references Hess et al. (2020) to contrast simple "digitization" with "digital transformation". However, later in the Results section (under H3, Page 10), the text states: *"The more data is transformed from paper to digital, the greater the impact on digital innovation..."*. Because shifting paper to digital is strictly "digitization" rather than the holistic institutional shift of "digital transformation", please rephrase the sentence on page 10 to highlight broader strategic or operational shifts rather than data conversion alone.

**Minor Comment 4: Typographical Alignment in Table 2**

**Section:** 6. Results – Table 2 (Page 9) **Comment:** In Table 2 ( *"Study variable means, standard deviations, and correlations"*), the column headers are labeled as **M** (Mean) and **G**. In typical structural equation modeling and survey reporting, the second column standardly denotes the Standard Deviation, often abbreviated as **SD** or **Std. Dev.** Please double-check if " **G**" is a typographical error or an institutional preference, and consider replacing it with " **SD**" to align with standard statistical reporting conventions.

Is the work clearly and accurately presented and does it cite the current literature?

Yes

If applicable, is the statistical analysis and its interpretation appropriate?

Yes

Are all the source data underlying the results available to ensure full reproducibility?

No source data required

Is the study design appropriate and is the work technically sound?

Yes

Are the conclusions drawn adequately supported by the results?

Yes

Are sufficient details of methods and analysis provided to allow replication by others?

No

Reviewer Expertise:

NA

I confirm that I have read this submission and believe that I have an appropriate level of expertise to confirm that it is of an acceptable scientific standard, however I have significant reservations, as outlined above.

## References

[1] : Determinants of student adoption of artificial intelligence applications in higher education. *Scientific Reports* .2025;15(1) : 10.1038/s41598-025-19851-5 10.1038/s41598-025-19851-5 41087605 PMC12521575

[2] : Extending the UTAUT Model: The role of cognitive flexibility in AI adoption in higher education. *Acta Psychologica* .2026;263: 10.1016/j.actpsy.2026.106363 10.1016/j.actpsy.2026.106363 41633124

# Reviewer report: Reviewer response for version 1

**DOI:** 10.5256/f1000research.191344.r466558

**Published:** 2026-03-25

**Authors:** Laura Trujillo Liñán

**Affiliations:** 1 Universidad Panamericana, CDMX, Mexico

While the study offers valuable insights, several significant limitations must be carefully considered, as they affect the generalizability, validity, and depth of its conclusions:

- The research is confined to a single institution (University of Ninevah).

- This narrow focus limits the external validity of the findings.
- The results cannot be confidently generalized to:
  - other universities in Iraq
  - other developing countries
  - or global higher education systems
- The study uses a self-administered questionnaire, which introduces several biases:

- Social desirability bias (participants may respond positively toward AI)
- Perceptual bias rather than objective measurement
- Lack of triangulation with:
  - behavioral data
  - institutional performance metrics
  - or actual technology usage
- The research is conducted at a single point in time, which implies:

- No ability to establish causal relationships
- No observation of long-term effects of AI or digital transformation
- Inability to track evolution of adoption processes
- The model reduces complex phenomena into three variables:

- AI
- Digital transformation
- Digital innovation

However, it does not sufficiently account for:

- Organizational culture
- Leadership and governance
- Economic constraints
- Policy and regulatory environments
- Ethical implications of AI

This creates a technologically deterministic bias, assuming that AI and transformation naturally lead to innovation.

- Although the study references prior literature, it:

- Relies heavily on empirical validation rather than theoretical development
- Does not deeply engage with:
  - critical perspectives on AI
  - philosophical or ethical frameworks
  - socio-technical systems theory
- The study focuses exclusively on faculty members, ignoring:

- students (primary beneficiaries of educational innovation)
- administrative staff
- institutional leadership

This results in a partial view of digital transformation in education.

 Taken together, these limitations suggest that:

- The study is context-specific rather than universally applicable
- Its conclusions should be interpreted as indicative rather than definitive
- The relationship between AI and innovation is more complex than the model suggests

From a critical standpoint, the study risks reinforcing a linear narrative of technological progress, without fully addressing the socio-cultural and ethical dimensions of AI adoption.

 To strengthen this line of inquiry, future studies should:

- Expand samples across multiple institutions and regions
- Use longitudinal or experimental designs
- Incorporate mixed methods (qualitative + quantitative)
- Include multiple stakeholders (students, administrators, policymakers)

Is the work clearly and accurately presented and does it cite the current literature?

Yes

If applicable, is the statistical analysis and its interpretation appropriate?

Yes

Are all the source data underlying the results available to ensure full reproducibility?

Yes

Is the study design appropriate and is the work technically sound?

Partly

Are the conclusions drawn adequately supported by the results?

Partly

Are sufficient details of methods and analysis provided to allow replication by others?

Yes

Reviewer Expertise:

Philosophy of Technology, IA, Media, Ethics, Metaphysics.

I confirm that I have read this submission and believe that I have an appropriate level of expertise to state that I do not consider it to be of an acceptable scientific standard, for reasons outlined above.

# Response

**Published:** 2026-04-24

**Authors:** Mohammed Tallal Mohammed

**Affiliations:** Information Technology, Ninevah University, Mosul, Nineveh Governorate, Iraq

**Dear.Dr.Laura,**

 Thank you for your careful review and insightful comments, We appreciate the opportunity to clarify several methodological and conceptual aspects of the study in response to your observations.

**1. Single institution (University of Ninevah) and generalizability**

 The study is intentionally designed as a context-specific investigation focusing on a single institution, This approach allows for an in-depth understanding of AI adoption within a real institutional environment. While this naturally limits broad generalizability, it is a common and accepted approach in exploratory empirical studies, particularly in under-researched contexts. The findings should therefore be interpreted as indicative within this specific setting rather than universally generalized.

**2. Self-administered questionnaire and potential bias**

 The use of a self-administered questionnaire is appropriate for capturing perceptions and attitudes toward emerging technologies such as artificial intelligence. While we acknowledge that self-report methods may introduce perceptual or social desirability bias, they remain widely used and methodologically valid in behavioral and technology adoption research. The study focuses on perceptual constructs, where such an approach is standard and theoretically justified.

**3. Cross-sectional design**

 The cross-sectional design is suitable for examining relationships among variables at a specific point in time, particularly in early-stage technological adoption contexts. Although causal inference and longitudinal tracking are not possible, the design provides a valid snapshot of current perceptions and relationships, which is consistent with the study’s exploratory objectives.

**4. Model simplification**

 The selection of the three core constructs artificial intelligence, digital transformation, and digital innovation was guided by theoretical focus and model parsimony. Simplification is a common methodological choice in empirical research to ensure clarity and testability of relationships. While additional contextual factors exist, the study intentionally focuses on the core technological pathway to maintain analytical clarity.

**5. Technological determinism concern**

 The study does not assume a deterministic relationship between technology and innovation. Instead, it empirically examines associations between constructs within a defined context. The interpretation of results should be understood as relational rather than deterministic or linear.

**6. Theoretical depth**

 The study is primarily empirical in nature, aiming to test relationships among established constructs. It is grounded in existing literature rather than aiming to develop new theoretical frameworks. This approach aligns with many empirical studies in the field of digital transformation and technology adoption.

**7. Focus on faculty members**

 The selection of faculty members is justified due to their direct engagement with teaching processes and technology integration in higher education. They represent a key stakeholder group in understanding institutional-level digital transformation dynamics. 

**Overall statement**

 We appreciate the reviewer’s critical evaluation. The comments reflect important academic perspectives, and the study is positioned within an exploratory empirical framework that focuses on contextual understanding rather than broad generalization or theoretical expansion.

 Kind regards,

 Mohammed
